# Disease-Specific Novel Role of Growth Differentiation Factor 15 in Organ Fibrosis

**DOI:** 10.3390/ijms26125713

**Published:** 2025-06-14

**Authors:** Harshal Sawant, Alip Borthakur

**Affiliations:** Department of Biomedical Sciences, Joan C. Edwards School of Medicine, Marshall University, Huntington, WV 25701, USA; sawantha@marshall.edu

**Keywords:** Growth Differentiation Factor 15, organ fibrosis, inflammation, TGF-β, extracellular matrix, macrophage polarization

## Abstract

Growth Differentiation Factor 15 (GDF15), also known as non-steroidal anti-inflammatory drug-activated gene-1 (NAG-1) or macrophage inhibitory cytokine 1 (MIC-1), is a stress- and inflammation-induced cytokine distantly related to the TGF-β superfamily. Its highly elevated levels showed close association with various pathological conditions, making it an emerging biomarker of disease prognosis. However, most GDF15-mediated effects under normal physiology and various pathological conditions are poorly understood. This is partly because the only known GDF15 receptor is exclusively localized in the brain, and how GDF15 functions peripherally is currently unknown. Mounting recent evidence has shown GDF15’s critical role in fibrosis in multiple organs, such as the liver, lung, and kidney. Evidence further suggests that it can either contribute to fibrosis by promoting inflammation and fibroblast activation or confer protective effects by modulating the immune response and mitigating fibrosis severity. Thus, the exact role of GDF15 in fibrosis can vary depending on the organ involved and the specific disease context. For example, increased GDF15 in idiopathic pulmonary fibrosis (IPF) promotes fibrosis via fibroblast activation and collagen deposition. Conversely, GDF15 might have a protective role in liver fibrosis, with decreased GDF15 levels causing increased fibrosis severity, while GDF15 treatment ameliorates fibrosis. Due to its close association with fibrosis, GDF15 is being investigated as a potential biomarker for disease severity and monitoring treatment response. However, further research unraveling its mechanisms of action is needed to explore the potential of GDF15 as a therapeutic target for treating fibrosis, either by modulating its expression or utilizing its immunomodulatory properties. This review marshals the limited studies addressing the recently appreciated differential role of GDF15 in regulating the fibrotic process in different organs. The review also discusses the aspects of further research needed to highlight GDF 15 as a novel predictor and therapeutic target for fibrosis in different organs.

## 1. Introduction

**Figure 1:**
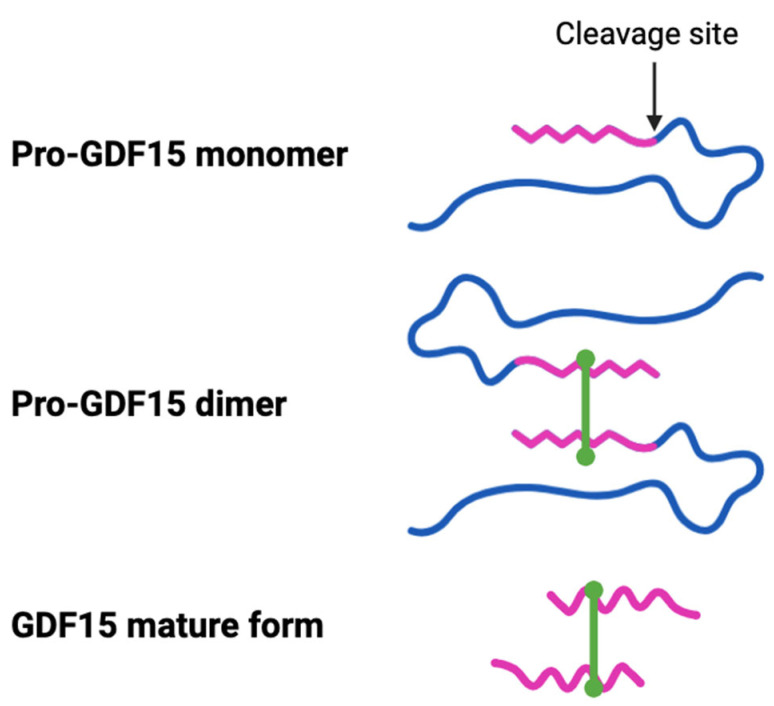
The precursor GDF15 propeptide is proteolytically cleaved at an N-terminal site to form a mature protein (25 kDa). Two mature proteins linked through a disulfide bond form the active circulating homodimer GDF15 protein. The excised (blue) and retained (pink) parts of the propeptide are shown in different colors.

GDF15, also known as macrophage inhibitory cytokine-1 (MIC-1), prostate-derived factor (PDF), nonsteroidal anti-inflammatory drug (NSAID)-activated gene-1 (NAG-1), placental bone morphogenetic protein (PLAB), and placental transforming growth factor (PTGF), is a divergent member of the transforming growth factor-β (TGF-β) superfamily [1]. In humans, the *GDF15 gene* located on chromosome 19p13.11 encodes a ~40 kDa precursor protein consisting of 308 amino acids [2,3]. This propeptide is proteolytically cleaved at an N-terminal site to form a mature protein (25 kDa). Two mature proteins linked through a disulfide bond form the active circulating homodimer GDF15 protein [4,5] (Figure 1). GDF15 is soluble and circulates in the bloodstream at 0.15–1.15 ng/mL serum levels under normal physiological conditions [6]. Elevated levels of GDF15 are linked to pathological conditions, including tissue damage, inflammation, infection, as well as the development of cardiovascular diseases, endocrine diseases (diabetes and obesity), and cancer [5]. Given its diverse range of functions in various cellular processes in different tissues, it is critical to understand the mechanisms by which GDF15 acts, the receptors with which it interacts, and the upstream and downstream signaling events involved. However, there is a significant gap in knowledge of the pathways induced in specific cell types or disease states. Numerous clinical studies link elevated GDF15 to the progression and severity of several pathologies, including fibrosis progression in different organs. Similarly, multiple studies in experimental models have described GDF15-dependent effects in normal physiology and disease states; however, the mechanisms of these effects are complex, diverse, inconsistent, and not well defined, and therefore, lack any clear consensus [3,5].

Tissue fibrosis is a pathological scarring process associated with excessive deposition of extracellular matrix (ECM) that leads to the destruction of organ architecture and impairment of organ function [7]. Excessive ECM is produced by the increased number of mesenchymal cells, including fibroblasts, myofibroblasts, and smooth muscle cells [8,9]. In the normal wound healing process following tissue injury, fibroblasts are transiently activated, proliferate, and transform into myofibroblasts to produce higher levels of ECM components. However, dysregulation of the homeostatic wound healing and tissue repair leads to perpetual fibroblast activation and sustained accumulation of ECM. Further, under normal physiology, once the healing process is accomplished, the fibrotic complex is degraded by matrix metalloproteases (MMPs), and myofibroblasts undergo apoptosis or revert to a non-activated state [10]. However, recurrent or persistent tissue injury and inflammation sustain ECM deposition and fibrogenesis, which can progress even after the inflammatory trigger has subsided. Therefore, despite inflammation being a key causative factor of fibrogenesis, anti-inflammatory drugs were ineffective in ameliorating fibrosis, suggesting that fibrotic progression mechanisms could be distinct and partly independent of inflammation.

Although elevated circulatory levels of GDF15 have been associated with chronic inflammatory conditions in various renal, lung, liver, and cardiovascular diseases [11], its involvement in organ fibrosis began to emerge only in recent studies during the past few years. First discovered in 1997, GDF15 was known as macrophage inhibitory cytokine (MIC) due to its role in inhibiting macrophage activation [2]. Later research, particularly the studies published during the late 2010s and early 2020s, revealed more complex roles of GDF15, including its involvement in fibrotic processes in different organs. However, depending on the organ and the specific fibrotic condition, GDF15 has been shown to aggravate, and conversely, inhibit fibrotic progression. In vivo studies, particularly using bleomycin-induced lung fibrosis models, demonstrated that GDF15 neutralization reduced collagen deposition and attenuated fibrotic development [12]. On the other hand, GDF15 has been shown to exert both profibrotic and antifibrotic effects in fibrogenesis associated with various diseases of the liver, kidney, and heart [13,14,15,16,17,18,19,20]. Therefore, despite multiple studies providing convincing evidence of a link between GDF15 and specific fibrotic conditions, an increased understanding of the mechanisms of its effects is needed to highlight GDF15 as a novel therapeutic target for fibrotic diseases.

In this review, we attempt to compile the clinical, translational, and experimental animal model studies that have substantially contributed to our current knowledge of the complex role of GDF15 in organ fibrosis. We also discuss further studies needed to fill the gaps towards establishing GDF15 as a novel target to develop anti-fibrotic therapy.

## 2. Cellular and Molecular Mechanisms of Tissue Fibrosis

Tissue fibrosis occurs due to excessive deposition of extracellular matrix (ECM) proteins, primarily collagen, following the activation of fibroblasts into myofibroblasts [21]. Initial tissue damage triggers an inflammatory response by recruiting immune cells like macrophages, which release cytokines like TGF-β, promoting the transition of local fibroblasts into myofibroblasts, the central players in fibrosis. The myofibroblasts are differentiated fibroblasts expressing α-smooth muscle actin (α-SMA), significantly enhancing collagen production [7,22]. TGF-β, which is considered another master regulator of fibrosis, stimulates the activation of fibroblasts into myofibroblasts, promotes collagen synthesis, and inhibits collagen degradation. In certain situations, epithelial cells undergo a process called epithelial-to-mesenchymal transition (EMT), transforming into mesenchymal cells with fibroblast-like characteristics and contributing to the myofibroblast pool. Chemokines released by inflammatory cells attract additional fibroblasts and immune cells to the site of injury, perpetuating the fibrotic process. The deposited collagen fibers can undergo further modifications, including cross-linking, leading to a dense and rigid fibrotic scar [23]. Chronic inflammation, oxidative stress, and multiple growth factors are key triggers of tissue fibrosis. Persistent inflammatory stimuli, such as autoimmune diseases or chronic infections, can drive fibrosis. Reactive oxygen species (ROS) can activate signaling pathways that promote fibroblast activation and collagen production. Besides TGF-β, other growth factors like platelet-derived growth factor (PDGF) can also contribute to fibrogenesis [24]. Certain genetic variations can predispose individuals to excessive fibrosis in specific organs.

## 3. Chronic Inflammation: Primary Causative Factor, but Not the Sole Driver of the Fibrotic Process

Fibrosis is the result of chronic inflammatory reactions induced by a variety of stimuli, including persistent infections, autoimmune reactions, allergic responses, chemical insults, radiation, and tissue injury [21]. The key cellular mediators of fibrosis are the myofibroblasts, which, when activated, serve as the primary collagen-producing cells. Myofibroblasts are generated from a variety of sources, including resident mesenchymal cells, epithelial and endothelial cells in processes termed epithelial/endothelial-mesenchymal (EMT/EndMT) transition, as well as from circulating fibroblast-like cells called fibrocytes that are derived from bone marrow stem cells. Myofibroblasts are activated by a variety of mechanisms, including paracrine signals derived from lymphocytes and macrophages, autocrine factors secreted by myofibroblasts, and pathogen-associated molecular patterns (PAMPS) produced by pathogenic organisms that interact with pattern recognition receptors (i.e., TLRs) on fibroblasts. Cytokines (IL-13, IL-21, TGF-beta1), chemokines (MCP-1, MIP-1beta), angiogenic factors (VEGF), growth factors (PDGF), peroxisome proliferator-activated receptors (PPARs), acute phase proteins (SAP), caspases, as well as components of the renin–angiotensin–aldosterone system—Angiotensin II (ANG II) have been identified as important regulators of fibrosis and are being investigated as potential targets of antifibrotic drugs [21].

While prolonged tissue inflammation is a prerequisite for fibrogenesis, controlling inflammation alone cannot stop or reverse the fibrotic process, suggesting that inflammation is not the sole driver of fibrosis [25]. Although current treatments for fibrotic diseases such as idiopathic pulmonary fibrosis, liver cirrhosis, systemic sclerosis, progressive kidney disease, intestinal fibrosis, and cardiovascular fibrosis typically target the inflammatory response, there is accumulating evidence that the mechanisms driving fibrogenesis are distinct from those regulating inflammation. Indeed, some studies have suggested that ongoing inflammation is needed to reverse established and progressive fibrosis. Therefore, identifying critical signaling component(s) that trigger and sustain the fibrotic process irrespective of the inflammatory state is paramount to identifying novel anti-fibrotic therapy targets.

## 4. GDF15: An Inflammation and Stress-Associated Cytokine with Poorly Defined Biology

GDF15, also known as macrophage inhibitory cytokine 1 (MIC-1) or NSAID-activated gene 1 (NAG-1), belongs to the TGF-β superfamily with a poorly understood biology, playing roles in inflammation, tissue damage, and metabolic regulation. It is a stress-induced cytokine, meaning its expression and release are upregulated in response to various stress signals, including inflammation, tissue injury, and hypoxia. Intracellularly, GDF15 undergoes dynamic trafficking between the cytoplasm, nucleus, and extracellular matrix (ECM) [5]. Under normal physiological states, GDF15 mRNA is produced at low levels in various cells and tissues, including the kidney, lung, pancreas, heart, skeletal muscle, adipose tissue, liver, gastrointestinal tract, placenta, and brain. In humans, GDF15 protein expression is notably high in the placenta, moderate in the prostate and urinary bladder, and low in the gastrointestinal tract, pancreas, and kidney [1,26,27,28]. In response to inflammatory stimuli, GDF15 is primarily produced by macrophages [29,30]. The role of GDF15 in the macrophage system has been increasingly investigated in recent years. Macrophages produce high levels of GDF15 during oxidative and lysosomal stress, which can lead to fibrogenesis and angiogenesis at the tissue level. At the same time, macrophages can respond to GDF15 by switching their phenotype to a tolerogenic one [29]. Elevated GDF15 levels are linked to pathological conditions, including tissue damage and inflammation, as well as to the development of cardiovascular diseases, metabolic diseases, and cancer. Given its diverse functions in different tissues and cellular processes, it is critical to understand the process by which GDF15 acts, the receptors with which it interacts, and the resulting signaling events involved. Unfortunately, a significant gap exists in understanding which pathways are activated in specific cell types or conditions.

## 5. Emerging Role of GDF15 in Modulating Fibrosis in Different Organs

GDF15 plays a complex role in organ fibrosis, acting either as a pro-fibrotic or anti-fibrotic factor in various organs, such as the liver, lung, and kidney. In some cases, it is linked to disease severity, and its neutralization has been shown to inhibit the fibrotic process in specific experimental models. GDF15 can activate pathways that promote fibrotic processes, such as the TGF-β signaling pathway. It can also interact with other factors involved in fibrosis, like macrophages and fibroblasts. However, due to its opposite role in modulating fibrosis in different organs and different contexts, further research unraveling its mechanisms of action is needed to explore the potential of GDF15 as a therapeutic target for treating fibrosis. The following sections discuss the studies reported in the literature about the pro-fibrotic and anti-fibrotic effects of GDF15 (Table 1) in the liver, lung, kidney, heart, and intestinal diseases.

### 5.1. Pro- and Anti-Fibrotic Role of GDF15 in Liver Diseases

GDF15 is implicated in liver fibrosis, both positively and negatively, depending on the context and specific mechanisms involved. While some studies suggest GDF15 can ameliorate liver fibrosis by modulating liver macrophages and potentially inhibiting TGF-β signaling, others indicate it can promote fibrosis progression. Differential effects of GDF15 in liver fibrosis are discussed below by citing four clinical studies and two experimental animal models.

In metabolic dysfunction-associated steatotic liver disease (MASLD), elevated serum levels of GDF15 were positively correlated with the severity of hepatic inflammation and fibrosis [13,14]. These clinical cohort studies defined GDF15 as a novel non-invasive index of fibrotic progression in liver cirrhosis. Other human studies in non-alcoholic fatty liver disease (NAFLD) have provided evidence that GDF15 levels are significantly associated with advanced fibrosis and could also be potentially involved in linking type 2 diabetes and fibrosis in NAFLD [13,36]. The lack of mechanistic explanations in these human studies does not preclude using GDF15 as a potential biomarker of fibrosis. However, further large-scale cohort studies with a prospective longitudinal design and unraveling the causal relationship between GDF15 and fibrosis progression are warranted to confirm the clinical usefulness of GDF15 as a novel biomarker for predicting advanced fibrosis in liver diseases. In mouse models and in vitro studies, GDF15 has been shown to promote hepatic stellate cell activation and liver fibrosis progression by regulating the TGF-β signaling pathway [37]. In contrast to these reports, a recent study with human subjects and animal models has shown that GDF15 ameliorates liver fibrosis by metabolic reprogramming of macrophages, major players of the fibrotic process [15]. Both in human patients and animal models, GDF15 expression was shown to be decreased in the fibrotic/cirrhotic liver compared with those without liver disease. Mechanistically, as shown in the animal models, GDF15 exerted its effects by reprogramming the metabolic pathways of macrophages to acquire an oxidative phosphorylation–dependent anti-inflammatory functional fate. Furthermore, the adoptive transfer of GDF15-preprogrammed macrophages to mouse models of liver fibrosis induced by CCl4 attenuated inflammation and alleviated the progression of liver fibrosis [15].

Multiple potential factors related to the study designs could influence the study results of the GDF15 effects on fibrosis. For instance, both the studies mentioned above [15,37] utilized CCl4-induced fibrosis in animal models. However, the CCl4 dose used by the former study [37] was substantially higher (0.5 L/g body weight) compared to the latter study [15] (1 mL/Kg body weight), which appears to be the most likely cause of the opposite effects of GDF15 (pro- or antifibrotic). Further, the opposite effects of GDF15 (pro- versus anti-fibrotic) in clinical versus pre-clinical (animal or in vitro) studies could be because the normal circulatory levels of GDF15 are different in humans and rodents. Moreover, the dose of recombinant GDF15 (rGDF15) used in vitro or administered to animals is often much higher than the normal physiological levels.

### 5.2. Role of GDF15 in Pulmonary Fibrosis

Pulmonary fibrosis is a devastating chronic lung disorder characterized by fibroblast accumulation and extracellular matrix deposition in the lung, with limited therapeutic options. The different types of pulmonary fibrosis are subgroups of interstitial lung diseases (ILDs). They are associated with a chronic and often progressive course [42]. The most common type of pulmonary fibrosis is idiopathic pulmonary fibrosis (IPF). Among other relevant types, the most important ones are fibrosing hypersensitivity pneumonitis (fHP) and ILDs associated with systemic diseases, all of which are rare and generally carry a poor prognosis [42]. The role of GDF15 in the progression of lung fibrosis has been emphasized in multiple recent studies in humans and experimental animal models. Both pro- and antifibrotic roles of GDF15 are exemplified in the following discussion, citing four clinical and three animal model studies.

Hypersensitivity pneumonia (HP) is an immune-mediated ILD that may be fibrotic (fHP) or non-fibrotic (non-fHP). Fibrosis in HP is associated with poor prognosis, emphasizing the need for biomarkers to distinguish fHP from non-fHP. In this regard, a recent human study revealed significantly elevated GDF15 levels in fHP compared to non-fHP, suggesting GDF15 is a valuable marker to distinguish between the two forms of HP [43]. The most common and devastating ILD is idiopathic pulmonary fibrosis (IPF) characterized by a progressive decline of respiratory function and early mortality [44]. Some IPF patients may suffer an acute, clinically significant, respiratory deterioration with new widespread alveolar abnormalities, referred to as acute exacerbation of IPF (AE-IPF), which has a poorly defined etiology [45]. A human study has shown that serum GDF15 levels and GDF15 mRNA and protein levels were significantly elevated in AE-IPF patients compared to healthy controls. The serum GDF15 level was correlated with the clinical variables of inflammation, metabolism, and disease severity in IPF subjects. Further, the GDF15 serum concentration was significantly higher in decedents than in survivors [31]. In patients with IPF, GDF15 expression in lung tissue significantly increased and correlated with pulmonary function. Single-cell RNA sequencing of human lungs identified epithelial cells as the primary source of GDF15, and circulating concentrations of GDF15 were markedly elevated and correlated with disease severity and survival in multiple independent cohorts [32]. While clinical trials with patients suggested GDF15 as a novel predictive biomarker of lung fibrosis progression, contemporary mechanistic studies in vitro and animal fibrosis models showed that GDF15 facilitates lung fibrosis by activating macrophages and fibroblasts. GDF15 expression was upregulated in bleomycin-induced lung fibrosis model following the induction of senescence in alveolar epithelial cells and macrophages [33]. Increased GDF15 secretion by bleomycin-induced senescent alveolar cells, in turn, augmented pro-fibrotic M2 macrophages and fibroblast activation (increased α-smooth muscle actin) via the ALK5-Smad2/3 pathway [33]. Transcriptional profiling of senescent type II alveolar epithelial cells in bleomycin-challenged mice identified GDF15 as the most significantly upregulated secreted protein that was detected in peripheral blood and bronchoalveolar lavage [32]. GDF15 neutralization in bleomycin-induced lung fibrosis in mice ameliorated fibrosis, whereas recombinant GDF15 (rGDF15) stimulated α-smooth muscle actin expression in normal human lung fibroblasts [12]. These studies further implicate a potential direct role of GDF15 in stimulating lung fibrosis.

### 5.3. GDF15 Has Been Strongly Linked to Kidney Fibrosis

The studies investigating the association of GDF15 in kidney fibrosis are limited, found to be complex, and not fully understood. While some studies suggest a protective role of GDF15 in acute kidney injury (AKI) and certain types of chronic kidney disease (CKD), other research indicates that elevated GDF15 can be associated with an increased risk of developing or progressing kidney fibrosis, a hallmark manifestation in several types of progressive CKD [16]. One clinical trial and two animal studies are cited in the following discussion on the role of GDF15 in kidney fibrosis.

A clinical trial using two independent cohorts showed that circulating GDF15 levels strongly correlated with intrarenal expression of GDF15 and were significantly associated with increased risk of CKD progression and fibrosis [17]. However, other studies in vitro and using transgenic animal models showed opposite results, highlighting the protective role of GDF15 in kidney fibrosis. *Gdf15*-deficient mice developed more severe toxic acute kidney injury, while GDF15 overexpression or GDF15 administration was protective. Kidney expression of Klotho protein, a critical protective factor of kidney health [18], was severely decreased in *Gdf15*-deficient mice and was preserved by GDF15 overexpression or GDF15 treatment. Kidney fibrosis induced by unilateral ureteral obstruction was more severe in *Gdf15*-deficient mice, while GDF15 overexpression decreased kidney injury. GDF15 increased Klotho expression in vivo in healthy mice and in cultured tubular cells and prevented Klotho downregulation by inflammatory factors in tubular cells by inhibiting NF-ĸB activation [39,46]. In another study, ureteral obstruction-induced kidney fibrosis was alleviated following intraperitoneal injection of GDF15 peptides into mice [38]. Further, GDF treatments of primary fibroblasts isolated from the kidneys of these mice inhibited collagen production, thereby alleviating fibrotic progression.

### 5.4. GDF15 Association with Cardiac Fibrosis

The studies investigating GDF15’s role in cardiac fibrosis are very limited. GDF15 expression is highly induced in cardiomyocytes after ischemia/reperfusion and in the heart within hours after myocardial infarction (MI). However, the reason for this increased GDF15 production has not been well understood. Recent studies show associations between GDF15, inflammation, and cardiac fibrosis during heart failure and MI [47]. Limited studies (one clinical and two animal) on GDF15’s role in cardiac fibrosis are outlined below.

In an animal study, GDF15 was elevated in all myocardial cells during myocardial injury and might cause endothelial dysfunction in certain conditions, with deleterious consequences on cardiac function [48]. The potential association between elevated GDF15 and cardiac fibrosis during heart failure (HF) and MI, could presumably be via activation of TGF-β1, a potent stimulator of collagen-producing cardiac fibroblasts. In another human study, the relationship between GDF15 levels and atrial fibrosis in patients with atrial fibrillation (AF) and rheumatic heart disease (RHD) was examined. The plasma GDF15 levels and the GDF15 mRNA levels in atrial tissues were significantly higher compared to healthy subjects and positively correlated with the development and maintenance of atrial fibrosis [19]. However, the origin of this increased GDF15 pool in the heart and the mechanisms of the specific effects of GDF15 in heart fibrosis in these studies are not well defined. The scenario is further complicated by the fact that some other studies found protective effects of GDF15 on HF. In a rat model of HF, lentiviral-mediated silencing of GDF15 triggered myocardial fibrosis [increased α-smooth muscle actin (α-SMA) and excessive collagen deposition] compared to control, suggesting that GDF15 preserves cardiac function by inhibiting fibrosis progression [20].

### 5.5. Is GDF15 Linked to Intestinal Fibrosis?

Chronic inflammation and impaired wound healing trigger intestinal fibrosis, which is very common in inflammatory bowel disease (IBD) patients [49,50]. It occurs in both forms of IBD, namely, Crohn’s disease (CD) and ulcerative colitis (UC), although it is more prevalent in CD [51]. In most cases, fibrosis in CD that primarily occurs in the terminal ileum induces persistent luminal narrowing and strictures [52,53]. Although inflammation is the primary trigger of fibrogenesis, anti-inflammatory and immunosuppressive drugs were ineffective in controlling fibrosis progression and eliminating established complications [8,50]. There are only scarce recent reports about the role of GDF15 in IBD. A recent case-controlled study with IBD patients undergoing biologic therapy demonstrated consistently higher plasma levels of GDF15 in both CD and UC patients compared to healthy controls. The correlation analysis indicated significant relationships between GDF15 levels, patient age, fibrinogen, and IL-6 levels, highlighting GDF15 as a promising biomarker for severe IBD [40]. Another cross-sectional study also found increased plasma GDF15 levels in IBD patients compared to healthy controls, with no difference between CD and UC patients [41]. Based on serum GDF15 levels and correlation studies with epidemiological and disease characteristics (active, flares, remitting, relapsing), a recent study implicated GDF15 serum levels indicative of an index of disease activity and a prognostic index of disease progression for CD but not for UC [41]. However, no human or animal studies have investigated whether GDF15 plays a role in IBD-associated fibrosis. Since GDF15 has been shown to play a critical role in fibrosis in multiple organs, such as the liver, lung, heart, and kidney, studies investigating the potential role of GDF15 in intestinal fibrosis, a debilitating complication in IBD patients, are urgently needed. Gut dysbiosis (alteration of gut microbiota composition, diversity, and metabolism), a major environmental causative factor for IBD, has also recently been linked to the occurrence and progression of intestinal fibrosis. Indeed, adherent invasive *E. coli* (AIEC), a CD-associated pathogen, has been shown to have a strong association with fibrogenesis [54]. Whether AIEC or other IBD-linked pathogens regulate GDF15 or vice versa in fibrosis is not known and merits detailed investigation.

## 6. Knowledge Gap: Potential Mechanisms of GDF15 in Modulating Fibrosis

**Figure 2:**
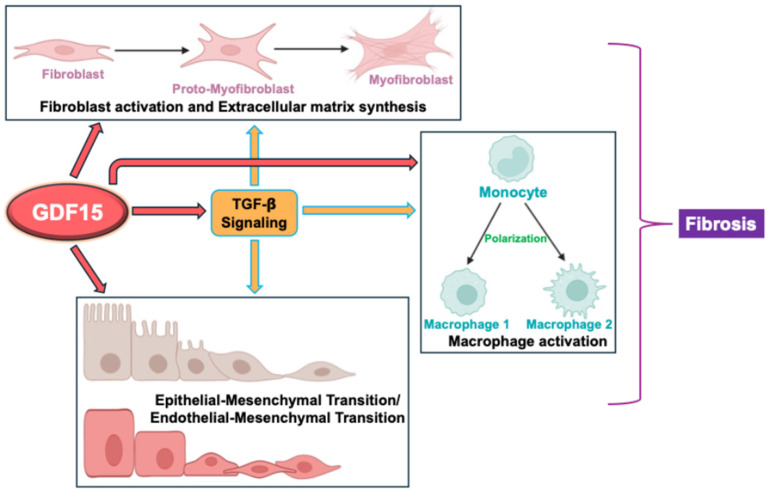
GDF15 modulates the fibrotic progression in different organs via its effects on (1) fibroblast activation to myofibroblasts and extracellular matrix deposition; (2) macrophage activation and polarization; and (3) epithelial–mesenchymal transition/endothelial–mesenchymal transition. GDF15 effects on these processes may be direct or via regulation of TGF-β signaling.

Despite its identification over 20 years ago, the functions of GDF15 remain complex and not fully elucidated. Its concentration in plasma varies widely depending on the physiological and pathophysiological state of the organism. However, most GDF15-mediated effects under normal physiology and various pathological conditions are poorly understood [55]. In particular, GDF15’s role in organ fibrosis is complex and not fully understood, but research suggests it can have both pro-fibrotic and anti-fibrotic effects, depending on the context. In some cases, it may contribute to fibrosis by stimulating fibroblast proliferation and activation through the paracrine TGF-β receptor pathway. However, in other instances, GDF15 may act as an anti-fibrotic agent by metabolic reprogramming macrophages, reducing inflammation, and inhibiting TGF-β signaling. As depicted in Figure 2 and described in the following sections, the mechanisms of GDF15’s role in fibrosis are context-specific and vary in different organs.

### 6.1. Myofibroblast Differentiation and Extracellular Matrix Formation

GDF15 can activate fibroblasts, leading to excess ECM production, a key characteristic of fibrosis. In bleomycin-induced lung fibrosis in mice, increased GDF15 secretion by senescent alveolar cells exerts pro-fibrotic effects by activating fibroblasts and macrophages [33]. Similarly, GDF15 was identified among the most significantly upregulated proteins in the IPF lung–derived ECM. In vivo, GDF15 neutralization in a bleomycin-induced lung fibrosis model attenuated fibrosis. In vitro, recombinant GDF15 stimulated αSMA expression in normal human lung fibroblasts (NHLF) via activin receptor-like kinase 5 (ALK5) receptor and reduced the migration of NHLF in collagen gel. These data suggest that GDF15 mediates lung fibrosis through fibroblast activation and differentiation, implicating a potential direct role of this matrix-associated cytokine in promoting aberrant cell responses in disease [12]. In recent studies, serum GDF15 levels were found to be significantly higher in patients with silicosis, a chronic fibrotic pulmonary disease caused by inhaling silica dust, a common mineral found in rock, sand, and clay. In vitro, GDF15 treatment activated human MRC5 lung fibroblast cells with upregulation of col1a and α-SMA through the miR-338/STAT1 pathway [56]. In another study, silica-stimulated senescence in alveolar epithelial cells secreted elevated levels of GDF15 that facilitated epithelial–mesenchymal transition (EMT) and fibroblast activation in a GDF15-dependent manner. Mechanistically, p53 regulated GDF15 transcription and secretion in senescence ATII cells. Moreover, secreted GDF15 performed its pro-fibrotic role by directly binding to TGF-βR via autocrine and paracrine manners. Also, lipid nanoparticles targeting GDF15 or cell senescence inhibitors NMN and BZBS showed efficient anti-fibrotic effects in vivo [35]. However, multiple other studies showed antifibrotic effects of GDF15 via inhibition of fibroblast activation. For example, GDF15 has been shown to repress TGF-β/SMAD signaling, leading to the inactivation of fibroblasts in pulmonary remodeling [34,57]. GDF15 has also been shown to ameliorate kidney fibrosis by inhibiting fibroblast growth and activation in vitro and in vivo [38]. In summary, GDF15 affects fibroblast activation and remodeling in a complex and context-dependent manner, either by promoting or inhibiting fibroblast activation depending on the specific cell type and disease state. The mechanisms and signaling pathways underlying GDF15 effects on fibroblasts are not fully known. Although the involvement of the TGF-β-SMAD pathway has been speculated in some studies, extensive mechanistic studies are needed to establish its context-specific opposite effects of fibroblast activation and inhibition during organ fibrosis.

### 6.2. Macrophage Polarization

Macrophages, key innate immune components, exhibit a high degree of plasticity and functional diversity and are critical for tissue homeostasis [58,59]. However, dysregulation of macrophages following tissue injury or chronic inflammation could contribute to intestinal fibrosis [60,61]. Stimulated by appropriate factors in the tissue microenvironment, macrophages undergo polarization to generate distinct subsets, such as classically activated M1 (pro-inflammatory) and alternatively activated M2 (anti-inflammatory/profibrotic) macrophages [62]. Growing evidence shows that macrophages, especially monocyte-derived M2 macrophages, play a pivotal role in fibrosis by secreting TGF-β1, the central protagonist of fibrosis [63] that prevents collagen degradation [64,65]. Further, macrophages can differentiate into collagen-producing myofibroblasts via macrophage-to-myofibroblast transition (MMT), promoting myofibroblast accumulation and producing excess ECM [66]. Profibrotic macrophages in intestinal fibrosis are mainly CD206+ M2a subtypes [60]. The role of GDF15 in the macrophage system has been increasingly investigated in recent years. Macrophages produce high levels of GDF15 during oxidative and lysosomal stress, which can lead to fibrogenesis and angiogenesis at the tissue level. At the same time, macrophages can respond to GDF15 by switching their phenotype to a tolerogenic one [29]. Various studies have shown that GDF15 can promote an anti-inflammatory phenotype in macrophages, reducing inflammation and potentially ameliorating fibrosis. However, GDF15 can also activate macrophages to a pro-fibrotic phenotype. A recent in vivo study showed that in *Gdf15* knockout mice, the intrahepatic microenvironment that developed during fibrosis showed relatively more inflammation due to enhanced infiltration of monocytes and neutrophils and increased expression of pro-inflammatory factors, which could be diminished by GDF15 overexpression in hepatocytes. GDF15 exerted its effects by reprogramming the metabolic pathways of macrophages to acquire an anti-inflammatory functional fate. Furthermore, the adoptive transfer of GDF15-preprogrammed macrophages to mouse models of liver fibrosis attenuated inflammation and alleviated fibrosis progression [15].

### 6.3. Epithelial-to or Endothelial-to-Mesenchymal Transition

In addition to the resident fibroblasts, the epithelial-to-mesenchymal transition (EMT) and endothelial-to-mesenchymal transition (EndMT), vital processes triggered by chronic inflammation and tissue injury, transform epithelial and endothelial cells into mesenchymal phenotype [67,68]. These EMT/EndMT-generated mesenchymal cells are an additional source of activated myofibroblasts to aggravate fibrosis via ECM deposition. GDF15 can exert both promoting and inhibiting effects on EMT/EndMT during organ fibrosis, depending on the context. During lung fibrosis, GDF15 secreted by the senescent alveolar type II epithelial cells promotes EMT of these cells via autocrine signaling through TGF-β receptors [35]. On the other hand, in a cell culture model, GDF15 significantly attenuated EMT markers in retinal pigment epithelial cells treated with EMT-inducing agents [69]. Therefore, GDF15’s influence on EMT and EndMT is still elusive, essentially needing further research to fully understand the complex role of GDF15 in EMT and EndMT during organ fibrosis.

### 6.4. Regulation of TGF-β Activity

GDF15 has been shown to affect organ fibrosis via modulation of both SMAD-dependent and independent TGF-β signaling mechanisms. Like other mechanisms of GDF15’s effects on fibrosis, it may activate or inhibit TGF-β signaling, respectively, to stimulate or ameliorate fibrosis progression. In a mouse model of liver fibrosis, activation of TGF-β signaling was shown to be the primary mechanism of the progressive effects of GDF15 in liver fibrosis [37]. However, another study showed increased liver fibrosis in *Gdf15*^−/−^ mice that also exhibited increased hepatic TGF-β activity and phosphorylated SMAD3. Recombinant GDF15 (rGDF15) subcutaneously injected in these mice reduced phosphorylated SMAD3 and attenuated fibrosis in the liver. Additional in vitro, in vivo, and human patient studies unraveling the context-specific complex role of GDF15 in modulating TGF-β signaling, a key process of fibrosis, are needed to understand the role of GDF15 in organ fibrosis.

### 6.5. Characterizing GDF15-Interacting Receptors in Peripheral Tissues

GDNF family receptor alpha-like (GFRAL), the only well-characterized receptor for GDF15, mediates its effects on food intake, body weight, and energy expenditure by activation of the GDF15-GFRAL-RET complex in neurons and axons in the hindbrain, where GFRAL is found to be exclusively localized [55]. The receptors mediating the effects of GDF15 in peripheral tissues, such as the modulation of fibrosis in different organs, are currently unknown. Since GFRAL-independent effects of GDF15 have also been shown in different cell types [70,71,72,73], additional receptors through which GDF15 mediates its effects, especially in peripheral tissues, have been postulated. An important factor while investigating GFRAL-independent effects of GDF15 in peripheral tissues is the GDF15 dose used in vitro. This is because, at very high concentrations, GDF15 may cross-react with another receptor in vitro that may not be relevant in vivo [54]. For example, recombinant GDF15 at 10 ng/mL used to treat human and mouse islets [70] was about 50–100 times higher than normal physiological circulating levels of GDF15. Other studies have also explored the possibility of GFRAL expression in locations other than the hindbrain [54]. A critical limitation of most of these studies, however, was the lack of proof of antibody specificity for GFRAL. Further research exploring GFRAL expression in peripheral tissues should emphasize the possibility that GFRAL is not expressed in most tissues under normal conditions, but, like GDF15, is triggered in specific cells or tissues by stress or disease conditions [54]. A novel experimental approach towards receptor validation for GDF15 will be to test rGDF15 or a GDF15 neutralizing antibody in peripheral tissues of a GFRAL knockout mouse model.

### 6.6. Exploring GDF15 as a Target for Anti-Fibrotic Drugs

The development of anti-fibrotic drugs involves targeting the mechanisms that lead to tissue scarring and damage, with the aim of retarding or reversing the progression of fibrosis. In this regard, a major hurdle in designing anti-fibrotic drugs is that the pathways leading to fibrosis are not currently fully understood. There has been an intense search for anti-fibrotic therapies over the last decade, and screening for their efficacy in preclinical models and clinical trials. Currently, two main anti-fibrotic drugs, nintedanib and pirfenidone, are approved for treating idiopathic pulmonary fibrosis (IPF), a progressive lung disease. Nintedanib inhibits multiple receptor tyrosine kinases, such as VEGF and PDGFR, which stimulate profibrotic pathways such as collagen synthesis, fibroblast activation, and EMT/EndMT processes. Pirfenidone, on the other hand, attenuates fibrotic progression by reducing inflammation and oxidative stress [74]. To highlight GDF15 as a novel target of anti-fibrotic drugs, the effects of these drugs on the circulating levels of GDF15 and its cellular functions need to be investigated. A recent study has shown that imperatorin, a naturally occurring furanocoumarin derivative, ameliorates lung fibrosis by stimulating GDF15 secretion in fibroblasts. Similar mechanistic studies are warranted to establish the role of GDF15 in mediating the effects of antifibrotic drugs, such as nintedanib and pirfenidone. A potential confounding factor in these studies could be that GDF15 exerts both pro- and antifibrotic effects depending on the context. Therefore, a clear mechanistic understanding of the role of GDF15 in organ fibrosis is a prerequisite to establishing GDF15 as a target of the existing and future anti-fibrotic drugs. This will also aid in the development of anti-GDF15 antibodies or agonists, depending on the disease context, to treat fibrosis in different organs.

## 7. Conclusions and Future Perspectives

In recent years, GDF15 has been increasingly recognized for its association with fibrosis in different organs, particularly in the liver and lungs, and in the heart and kidneys. GDF15 is often elevated in fibrotic tissues. However, its relationship with fibrosis and underlying mechanisms are not fully understood due to several factors. GDF15’s mechanisms of action in fibrotic progression are indeed complex, with evidence suggesting both pro-fibrotic and anti-fibrotic effects depending on the specific tissue and underlying disease. Further, GDF15’s effects on fibrosis are not simply a one-to-one interaction. Instead, it can induce different pathways and influence various cell types associated with fibrotic progression, making it difficult to clearly pinpoint a unifying mechanism of its influence on fibrosis. Another major factor for the limited understanding of the role of GDF15 in various tissues is that the receptors by which GDF15 binds to target cells are mostly unidentified. The GDNF α-like receptor (GFRAL) is the only known GDF15 receptor so far, the expression of which is limited to the area postrema and the nucleus of the solitary tract, two important hindbrain centers. GFRAL binds to GDF15 to mediate its effects in reducing food intake and body weight. To date, cognate receptor(s) for GDF15 that mediate its effects on peripheral tissues are currently unknown. GDF15 is emerging as a critical signaling molecule involved in the pathogenesis of inflammation, infection, stress, and immune imbalance-associated diseases, such as fibrosis, in multiple peripheral organs. Therefore, identifying novel GDF15 receptor(s) that mediate the effects of GDF15 in peripheral tissues will facilitate precise delineation of GDF15-induced signaling pathways in different tissues in the context of different diseases, including fibrosis. Another unexplored area of GDF15 links to fibrosis that needs priority research is its role in intestinal fibrosis, a common complication of inflammatory bowel diseases, including ulcerative colitis and Crohn’s disease. It has only recently been shown elevation of serum levels of GDF15 in IBD patients, but its significance to the development or prolongation of chronic inflammation has not been studied. In common with fibrosis in other organs, intestinal fibrosis also involves elevated mesenchymal cells, excessive deposition of collagen-rich ECM [8,9], and aberrant polarization of macrophages [60,61]. Therefore, in parallel with fibrosis in other organs, the demonstration of a definitive role of GDF15 in modulating fibroblast activation, ECM production, and macrophage polarization [12,33,37,75] in intestinal fibrosis will further help establish a consensus mechanism of GDF15’s involvement in organ fibrosis.

## Figures and Tables

**Table 1:** Context-specific pro-fibrotic (red) or anti-fibrotic (blue) effects of GDF15 depend on the specific tissues and the disease. MASLD: metabolic dysfunction-associated steatotic liver disease; RHD: rheumatic heart disease.

Organ/Tissues	Type of Study	Disease	Role of GDF15	Reference
Lung	Clinical/Animal	Idiopathic pulmonary fibrosis	Pro-fibrotic	[12,31,32]
	Animal	Bleomycin-induced experimental fibrosis	Pro-fibrotic	[33]
	Animal/In vitro	Bleomycin-induced experimental fibrosis	Anti-fibrotic	[34]
	Animal/In vitro	Silicosis of alveolar epithelial cells	Pro-fibrotic	[35]
Liver	Clinical	Non-alcoholic fatty liver disease	Pro-fibrotic	[13,36]
	Clinical	MASLD	Pro-fibrotic	[14]
	Animal	CCl4-induced experimental fibrosis in mice	Pro-fibrotic	[37]
	Animal	CCl4/DDC-induced fibrosis in mice	Anti-fibrotic	[15]
Kidney	Clinical	Chronic kidney disease	Pro-fibrotic	[17]
	Animal	Ureteral obstruction-induced fibrosis in mice	Anti-fibrotic	[38]
	Animal	Acute kidney injury-associated fibrosis	Anti-fibrotic	[39]
Heart	Clinical	Atrial fibrillation and RHD	Pro-fibrotic	[19]
	Animal	Rat model of heart failure	Anti-fibrotic	[20]
Intestine	Clinical	Inflammatory bowel diseases	Increased GDF15	[40,41]
